# Altered Intestinal Microbiota with Increased Abundance of *Prevotella* Is Associated with High Risk of Diarrhea-Predominant Irritable Bowel Syndrome

**DOI:** 10.1155/2018/6961783

**Published:** 2018-06-05

**Authors:** Tingting Su, Rongbei Liu, Allen Lee, Yanqin Long, Lijun Du, Sanchuan Lai, Xueqin Chen, Lan Wang, Jianmin Si, Chung Owyang, Shujie Chen

**Affiliations:** ^1^Department of Gastroenterology, Sir Run Run Shaw Hospital, Zhejiang University School of Medicine, Hangzhou, Zhejiang, China; ^2^Institute of Gastroenterology, Zhejiang University, Hangzhou, Zhejiang, China; ^3^Division of Gastroenterology, Department of Internal Medicine, University of Michigan Health System, Ann Arbor, MI, USA

## Abstract

Alterations in gut microbiota are postulated to be an etiologic factor in the pathogenesis of irritable bowel syndrome (IBS). To determine whether IBS patients in China exhibited differences in their gut microbial composition, fecal samples were collected from diarrhea-predominant IBS (IBS-D) and healthy controls and evaluated by 16S ribosomal RNA gene sequence and quantitative real-time PCR. A mouse model of postinfectious IBS (PI-IBS) was established to determine whether the altered gut microbiota was associated with increased visceral hypersensitivity. The results indicated that there were significant differences in the bacterial community profiles between IBS-D patients and healthy controls. *Prevotella* was more abundant in fecal samples from IBS-D patients compared with healthy controls (*p* < 0.05). Meanwhile, there were significant reductions in the quantity of *Bacteroides*, *Bifidobacteria*, and *Lactobacillus* in IBS-D patients compared with healthy controls (*p* < 0.05). Animal models similarly showed an increased abundance of *Prevotella* in fecal samples compared with control mice (*p* < 0.05). Finally, after the PI-IBS mice were cohoused with control mice, both the relative abundance of *Prevotella* and visceral hypersensitivity of PI-IBS mice were decreased. In conclusion, the altered intestinal microbiota is associated with increased visceral hypersensitivity and enterotype enriched with *Prevotella* may be positively associated with high risk of IBS-D.

## 1. Introduction

Irritable bowel syndrome (IBS) is a chronic functional GI disorder characterized by recurrent abdominal pain associated with altered bowel habits, either diarrhea (IBS-D), constipation (IBS-C), or both (IBS-M) [1]. IBS is a highly prevalent condition with a meta-analysis of 80 studies involving over 260,000 subjects showing a global prevalence of 11.2%, and IBS-D is the most predominant subtype of IBS [2]. The pathophysiology of IBS is likely heterogeneous and may involve abnormalities in GI motility, visceral hypersensitivity, gut barrier dysfunction, immune activation, low-grade inflammation and altered brain-gut communication [3, 4].

Humans are host to a diverse community of microbes collectively known as the human microbiota, of which the vast majority live in the gut [5]. Alterations in the gut microbiota have been postulated as a pathogenic mechanism leading to IBS. Emerging evidence suggests there may be differences in the microbiota of IBS patients and healthy controls. However, results about those differences are inconsistent and even contradictory sometimes [6]. Several studies have identified an abundance of Firmicutes, mainly Clostridium cluster XIVa and Ruminococcaceae, along with a reduction in the relative proportion of *Bacteroides* in IBS patients compared with healthy controls [7–10]. Depletion of *Bifidobacteria* has also been demonstrated in both fecal and mucosal samples of IBS patients [9, 11–13]. Furthermore, fecal transplants from IBS patients to germ-free mice leads to physiologic changes seen in IBS, including rapid GI transit, impaired gut barrier function, and visceral hypersensitivity [14]. These observations all support a causative role for gut dysbiosis in IBS. However, there are conflicting reports in studies about the composition of gut microbiota in IBS and there has not been a specific microbial signature identified in IBS to date. Moreover, the evidence currently is more descriptive than mechanistic and the mechanisms by which gut dysbiosis leads to IBS are unclear.

The diversity and composition of the gut microbiome vary depending on age, gender, cultural practices, geographic regions, and dietary patterns [15–17]. Therefore, Chinese IBS patients are likely to show significant differences in composition and diversity of their gut microbiome compared to Western populations. This may also significantly impact the ability to use gut microbial composition and function as potential bioassays as well as the possibility of influencing the gut microbiome for therapeutic effects in IBS. Although alterations in gut microbial composition in IBS patients based on high-throughput sequencing techniques have been identified in Western populations, there is little evidence for gut dysbiosis in non-Western populations.

This study seeks to characterize the fecal microbial composition in Chinese patients with diarrhea-predominant IBS (IBS-D) and identify differences with healthy controls. Secondly, we proposed using a well-established mouse model of IBS to determine causative effects of gut dysbiosis.

## 2. Materials and Methods

### 2.1. Subjects

Subjects who met Rome III criteria for IBS-D were recruited to participate in this study between March 2014 and December 2014. Healthy subjects without history of chronic diseases or gastrointestinal complaints were recruited as controls. Subjects who were pregnant, obese (BMI > 30), history of abdominal surgery or severe systemic diseases, or history of antibiotic or probiotic use within four weeks were excluded from the study. Each subject completed an enteric symptom questionnaire regarding IBS symptoms, including abdominal pain, pain frequency, stool character, stool urgency, passage of mucus, and abdominal distention. The Human Ethics Committee of Sir Run Run Shaw Hospital approved the study, and all subjects gave written informed consent.

### 2.2. Study Design

Six stool samples from IBS-D patients and healthy controls were randomly collected, and 16s rRNA MiSeq high-throughput sequencing was performed. Visceral sensitivity of each mouse was assessed by behavioral responses to colorectal distention (CRD), which was measured by a semiquantitative score abdominal withdrawal reflex (AWR).

### 2.3. DNA Extraction and PCR Amplification

Fresh stool samples were processed within 1 hour of collection from IBS-D patients and healthy controls. DNA was extracted by a QIAGEN stool kit (QIAGEN, Hilden, Germany) from 200 mg feces following the manufacturer's instructions with minor modifications. The V4-V5 region of the bacterial 16S ribosomal RNA gene was amplified by PCR (95°C for 30 s, 55°C for 30 s, and 72°C for 45 s and a final extension at 72°C for 10 min) using primers 338F 5′-barcode-ACTCCTACGGGAGGCAGCA-3′ and 806R5′-GGACTACHVGGGTWTCTAAT-3′ [18], where barcode is an eight-base sequence unique to each sample. PCR reactions were performed in triplicate 20 *μ*l mixture containing 4 *μ*l of 5x FastPfu buffer, 2 *μ*l of 2.5 mM dNTPs, 0.8 *μ*l of each primer (5 *μ*M), 0.4 *μ*l of FastPfu polymerase, and 10 ng of template DNA.

### 2.4. Illumina MiSeq Sequencing of Fecal Microbiota

Amplicons were extracted from 2% agarose gels and purified using the AxyPrep DNA Gel Extraction Kit (Axygen Biosciences, CA, USA) according to the manufacturer's instructions and quantified using QuantiFluor™ ST (Promega, USA). Purified amplicons were pooled in equimolar and paired-end sequenced (2 × 250) on an Illumina MiSeq platform (Majorbio Co. Ltd., Shanghai, China) according to the standard protocol. Raw fastq files were demultiplexed and quality-filtered using QIIME (version 1.17) with the following criteria: (1) the 250 bp reads were truncated at any site receiving an average quality score < 20 over a 10 bp sliding window, discarding the truncated reads that were shorter than 50 bp; (2) exact barcode matching, 2 nucleotide mismatch in primer matching, reads containing ambiguous characters were removed; and (3) only sequences that overlap longer than 10 bp were assembled according to their overlap sequence. Reads which could not be assembled were discarded. Operational taxonomic units (OTUs) were clustered with 97% similarity cutoff using UPARSE version 7.1, and chimeric sequences were identified. The phylogenetic affiliation of each 16S rRNA gene sequence was analyzed by RDP classifier against the silva (SSU115) 16S rRNA database using confidence threshold of 70%.

### 2.5. Quantitative Real-Time PCR

Based on the above results of 16S rRNA gene sequence or studies suggesting specific genes in bacteria related with IBS [9, 19], gene primers were designed for further study (Table 1). qPCR was performed with ROCHE LightCycler® 480 instrument (Rotor gene 6000 software, Sydney, Australia). SYBR Premix Ex Taq (Takara) was used to amplify the gene of specific bacterial groups. Each PCR was carried out in a final volume of 10 *μ*l, comprising SYBR® Green PCR master mixture, primers, and template DNA. The following thermal cycling parameters were used for amplification of DNA: reaction cycle at 95°C for 30 s followed by 40 cycles of initial denaturation at 95°C for 5 s and 20 s of annealing at 60°C. Quantitative analysis was done by using standard curves made from known concentrations of plasmid DNA containing the respective amplification for each set of primers. qPCR was run in triplicate for each sample. The numbers were converted to log_10_ for further statistical analysis.

### 2.6. Postinfectious IBS (PI-IBS) Mouse Model

3- to 4-week-old male NIH mice (Guangdong Medical Lab Animal Center, China) were housed in a sterile, pathogen-free, 25°C facility with a 12 h light/dark cycle and received standard diet and water ad libitum. Fourteen mice were randomly assigned to either the control or PI-IBS group and housed in the same environment for a week before the experiment was initiated. Mice in the PI-IBS group were infected with *Trichinella spiralis* larvae (350–400 larvae per mouse) by oral gavage (0.1 ml in 0.9% saline), while mice in the control group received the same volume of normal saline [20]. Animal experimental procedures were approved by the Animal Care and Use Committee of Sir Run Run Shaw Hospital.

Behavioral responses to colorectal distention (CRD) were assessed in all groups starting 8 weeks later by measuring the AWR using a semiquantitative score as described previously [21]. The anesthetized mouse was inserted an inflexible plastic balloon into the descending colon 2 cm from the anal verge and secured to the tail. The barostat balloon was connected to a manometer (range from 0 to 300 mmHg). And the pressure was controlled using syringe which also connected to the manometer. AWR was assessed during 20-second distention of balloon catheter followed by a 4-minute resting period. AWR was recorded during plastic balloon inflation to 20, 40, 60, and 80 mmHg. Balloon inflation was repeated three times for each value to achieve accurate results. A 5-point AWR score was obtained by visually grading behavioral response to different levels of CRD (0, the mice are in stable mood; 1, the mice are in unstable mood with twisting their heads; 2, contraction of abdominal muscles; 3, lifting of abdomen; and 4, body arching and lifting of pelvic structures). At the conclusion of the experiment, mice were sacrificed. Mouse jejunum, ileum, and colon were collected and stained with hematoxylin and eosin.

### 2.7. Fecal Bacteria in PI-IBS Mice

Fresh stool samples were collected from mice immediately after they were sacrificed. qPCR was used to detect the quantity of *Prevotella*, *Bifidobacterium*, *Lactobacillus*, and *Bacteroides* according to the methods described previously.

### 2.8. Cohousing with PI-IBS and Control Mice

Initially, PI-IBS and control mice were housed separately in different cages. To determine if abnormalities in fecal microbial composition seen in PI-IBS mice might be causative for visceral sensitivity and IBS, PI-IBS and control mice were transferred to one common cage with a 1 : 1 ratio. *Because mice would eat each other's feces, the gut microbial community of mice from the same cage would tend to be similar* [22, 23]. Fecal bacterial samples by qPCR and visceral sensitivity based on AWR to CRD scores were examined 8 weeks later.

### 2.9. Statistical Analysis

The criteria for valid reads of high-throughput sequencing were described above, and data analysis was carried out with R version 3.2.1. Quantity of 16S rRNA gene obtained from qPCR was calculated by absolute quantification and logarithms of the fecal 16S rRNA gene copy numbers. IBS subjects were compared with controls using Mann–Whitney *U* test. Data were expressed as mean ± standard deviation (SD). A value of *p* < 0.05 was considered statistically significant. Statistical analyses were performed with SPSS version 16.0.

## 3. Results

### 3.1. Subjects

Forty subjects (22 male, 18 female; mean age of 40.05 ± 13.26 years) meeting Rome III criteria for IBS-D were enrolled. Twenty healthy subjects (5 male, 15 female; mean age 46.45 ± 12.84 years) were recruited as controls. Enteric symptom questionnaire was used to assess IBS symptoms. IBS patients had significant clinical symptoms, including abdominal pain (92.5%), abdominal distention (45.0%), alteration in stool form (92.5%), stool urgency (35.0%), and passage of mucus (47.5%).

### 3.2. Characterization of Fecal Microbiota in IBS-D Patients

**Figure 1:**
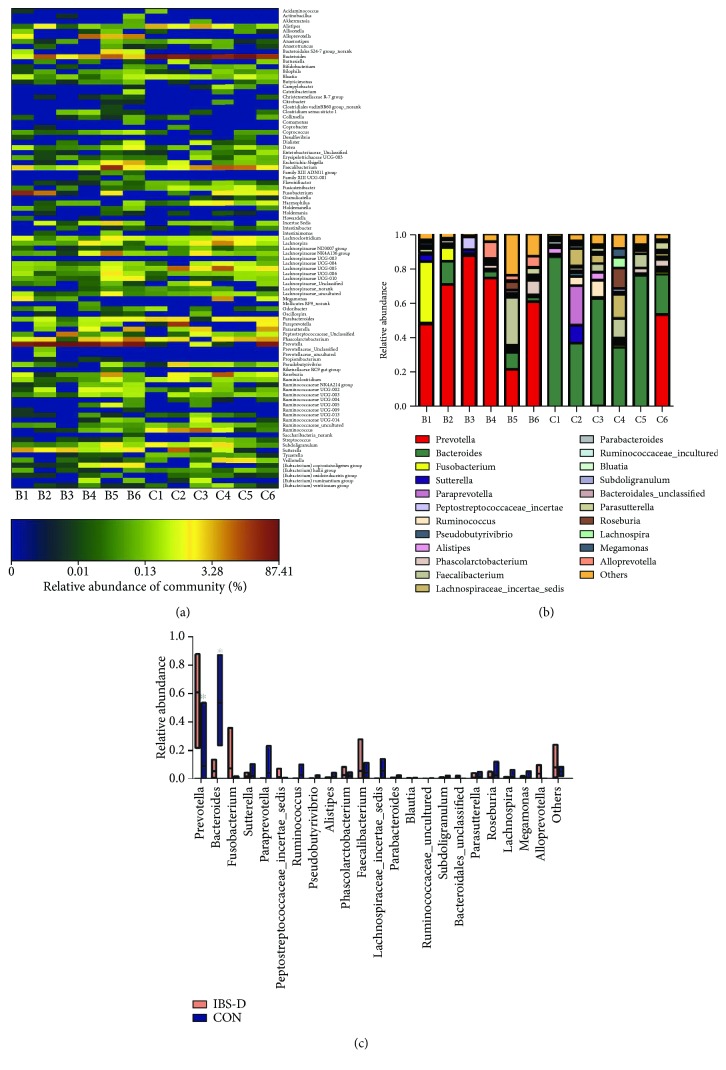
IBS-D patients show significant differences in fecal bacterial composition by high-throughput sequencing compared with healthy controls. (a) Heatmap of fecal microbiota in IBS-D patients and healthy controls. There are significant differences in bacterial community profiles at the genus level between IBS-D patients (*n* = 6) and healthy controls (*n* = 6). Each column represents one subject. C: control subjects; B: IBS-D patients. (b) Relative abundance of phylotypes at the genus level. (c) Differences in the relative abundance of phylotypes between IBS-D patients and healthy controls. IBS-D patients showed an abundance of *Prevotella* while *Bacteroides* predominates in healthy controls. ^∗^*p* < 0.05 versus control.

Six stool samples from IBS-D patients and healthy controls were collected and performed with 16s rRNA MiSeq high-throughput sequence. IBS-D patients and healthy subjects demonstrated significant differences in the bacterial community profiles at the genus level by heatmap analysis (Figure 1(a)). The level of specific bacteria in IBS-D also differed significantly from healthy controls. The relative abundance of *Prevotella* was the most striking alteration between the two groups. At the genus level, *Prevotella* was the dominant phylotype (60.53%) in IBS-D patients. Healthy controls meanwhile were predominated with *Bacteroides* phylotype (53.21%). The remaining genera including *Fusobacterium*, *Ruminococcus*, and *Sutterella* were not significantly different between IBS-D subjects and healthy controls (Figures 1(b) and 1(c)).

**Figure 2:**
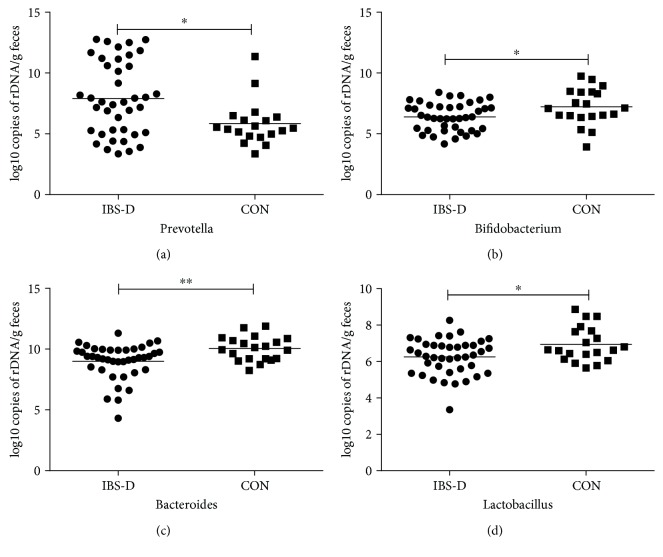
Quantity of different bacterial phylotypes in IBS-D and healthy controls (CON) measured by qPCR. IBS-D patients displayed a striking abundance of *Prevotella* while *Bacteroides*, *Bifidobacterium*, and *Lactobacillus* were significantly decreased in IBS-D compared with healthy controls. *p* values were calculated with the Mann–Whitney *U* test. ^∗^*p* < 0.05, ^∗∗^*p* < 0.01 versus the control group.

The quantity of bacteria in IBS-D patients and healthy controls was further analyzed by qPCR analysis. IBS-D patients again demonstrated a remarkable change in fecal microbial composition. The number of *Prevotella* in IBS-D patients was over 100-fold higher when compared with healthy subjects (*p* < 0.05) (Figure 2). Consistent with sequencing results, IBS-D patients demonstrated a significant decrease in the quantity of *Bacteroides* compared with healthy controls (*p* < 0.01). Fecal *Bifidobacteria* and *Lactobacillus* are significantly decreased in IBS-D patients compared to healthy controls (*p* < 0.05) (Table 2).

### 3.3. Altered Intestinal Microbiota Is Associated with Increased Visceral Hypersensitivity in PI-IBS Model

**Figure 3:**
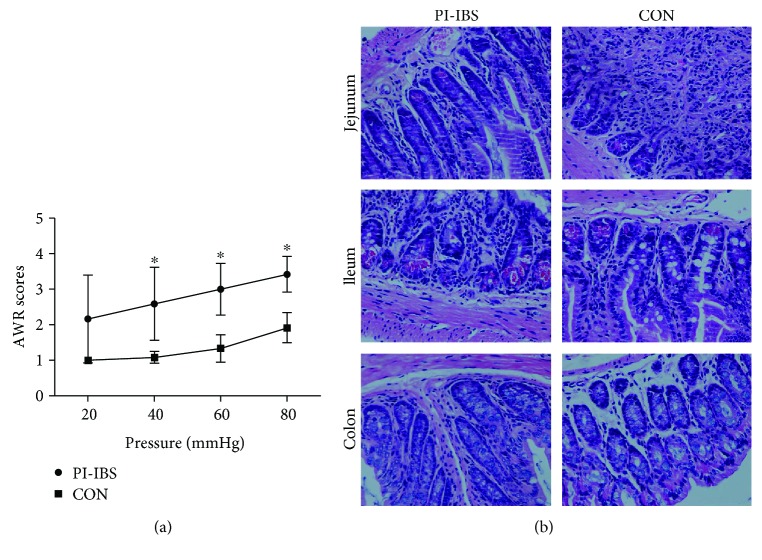
Relationship between AWR scores and histology in PI-IBS and control mice. (a) AWR scores to CRD. AWR scores at distention pressures of 40, 60, and 80 mmHg were significantly higher in PI-IBS mice than in control mice. (b) Hematoxylin and eosin (H&E) staining: representative sections of jejunum, ileum, and colon from PI-IBS or control mice (original magnification ×200). No evidence of inflammation, including neutrophil infiltration in the lamina propria or edema in interstitial tissues, was seen with PI-IBS mice compared with control mice. ^∗^*p* < 0.05 versus control.

We showed similar findings of fecal microbial composition using an established mouse model of PI-IBS. The abundance of fecal *Prevotella* is significantly increased by approximately 3-fold in PI-IBS mice compared with mice in the control group (*p* < 0.05). *Bacteroides*, *Bifidobacteria*, and *Lactobacillus* target bacteria did not show statistical differences between PI-IBS and control mice (Table 3), though there are trends towards a higher level of *Lactobacillus* in PI-IBS mice. We also demonstrated that PI-IBS mice had increased visceral sensitivity without obvious intestinal inflammation. AWR scores to CRD in PI-IBS mice were significantly higher compared with control mice at distention pressures of 40, 60, and 80 mmHg (*p* < 0.05) (Figure 3(a)). No significant pathological findings including hyperemia or edema were observed in PI-IBS mice (Figure 3(b)).

**Figure 4:**
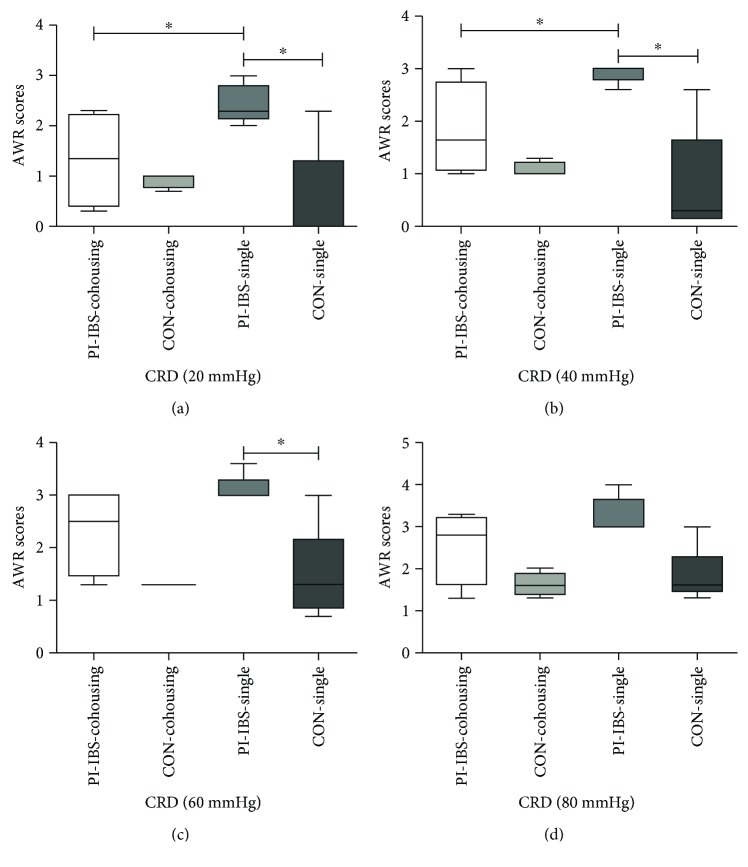
AWR scores of cohousing and single-housing groups. AWR scores at distention pressures of 20, 40, and 60 mmHg were significantly higher in PI-IBS mice that were single-housed (PI-IBS-single) compared with control mice (CON-single) as well as PI-IBS mice that were cohoused (PI-IBS-cohousing). Further, PI-IBS-cohousing mice showed no statistical differences compared with either control mice group. ^∗^*p* < 0.05 compared with controls. AWR: abdominal withdrawal reflex; CRD: colorectal distension.

After PI-IBS mice were cohoused with control mice, there was no statistical difference in the level of fecal *Prevotella* between the two groups. In addition, fecal *Prevotella* in cohoused mice showed no significant difference when compared with single-housed control mice. *Bacteroides*, *Bifidobacteria*, and *Lactobacillus* were equally contributed between single-housing and cohousing groups (Table 4). AWR scores to CRD in PI-IBS and control mice showed no significant difference when cohoused. Cohousing PI-IBS mice experienced decreased visceral hypersensitivity when compared to single-housed PI-IBS mice at distention pressures of 20 or 40 mmHg (*p* < 0.05) (Figure 4). These results demonstrate that cohousing PI-IBS mice normalizes the quantity of fecal *Prevotella* to levels similar to control mice and subsequently alleviates visceral hypersensitivity seen in PI-IBS.

## 4. Discussion

Recently, three distinct enterotypes have been identified, which are characterized by the dominant genera (*Bacteroides*, *Prevotella*, and *Ruminococcus*) [16, 17]. Previous studies have indicated conflicting results about the relationship between enterotype and IBS. Julien et al. reported that *Bacteroides*-dominant enterotype was more frequent in IBS subjects and *Prevotella*-dominant enterotype was more common in healthy subjects. The study also indicated that IBS symptom severity was associated negatively with enterotype enriched with *Prevotella* [24], while another study found that both *Bateroides*-dominant enterotype and *Prevotella*-dominant enterotype are associated with high risk of IBS-D and nondominant enterotype is more frequent in healthy subjects [25]. Our study also reported different result. The sequencing results indicated that *Prevotella* was the most dominant genera in IBS-D patients while *Bacteroides* was more frequent in healthy subjects. This was verified by qPCR analysis which demonstrated increased quantity of *Prevotella* and decreased quantity of *Bacteroides* in IBS-D patients. Our result was consistent with one study demonstrating that the level of *Prevotella* was increased in children diagnosed with IBS-D [26].

To further explore the change of intestinal microbiota and where altered intestinal microbiota is associated with increased visceral hypersensitivity in IBS-D, we established a *Trichinella spiralis*-induced PI-IBS model, which is more close to the type of IBS-D [27]. This model of PI-IBS also shows persistent disturbances in gut motility and visceral hypersensitivity [28, 29]. Interestingly, we also found significant increased quantity of *Prevotella* in PI-IBS mice. Because mice would eat each other's feces, cohousing mice from different groups would lead to transfer of gut microbiota from each other [22, 23]. So, we cohoused PI-IBS mice with control mice and found the PI-IBS mice exhibited decreased abundance of *Prevotella* and lower level of visceral hypersensitivity after cohousing. The decreased visceral hypersensitivity of PI-IBS after cohousing reflects that altered intestinal microbiota is associated with visceral hypersensitivity.

The consistent increased quantity of *Prevotella* in IBS-D patients and PI-IBS mice indicated that the enterotype enriched with *Prevotella* may be positively associated with high risk of IBS-D. This may be attributed to the following mechanism. Firstly, *Prevotella copri* has been indicated to possess a number of enzymes and gene clusters essential for fermentation and utilization of complex polysaccharides [30]. And *Prevotella* can positively interact with the other member of the community to promote increased carbohydrate fermentation [31]. Short-chain fatty acids (SCFAs) are one of the important by-products of carbohydrate fermentation, which have been reported to induce dose-dependent visceral hypersensitivity [32]. One study indicated that *Prevotella-*dominant enterotype induced higher SCFA production than *Bacteroides*-dominant enterotype. In addition, the fermentation of carbohydrates increases luminal H_2_ and CH_4_ production, resulting in luminal distention and pain in those with visceral hypersensitivity [33]. Therefore, *Prevotella* may interact with other microbiota to induce visceral hypersensitivity and exacerbate symptom of IBS by promoting carbohydrate fermentation. Secondly, Wright et al. demonstrated that *Prevotella* contains enzymes that are important in mucin degradation, which may lead to increased intestinal permeability. Thirdly, *Prevotella* has been associated with proinflammatory function. Treatment mice with *Prevotella copri* exacerbate colitis induced by dextran sulfate sodium [34]. Dillon et al. [35] suggested that increased levels of *P. copri* might contribute to driving chronic inflammation in individuals infected with HIV. Furthermore, Lukens et al. demonstrated gut dysbiosis with abundance of *Prevotella* in a mouse model of osteomyelitis [36].

We also demonstrated lower level of *Bifidobacterium* and *Lactobacillus* in IBS-D patients. *Bifidobacterium* and *Lactobacillus* are both abundant commensal flora in the human intestine and may play a protective role in maintaining gut integrity [37]. It is certainly plausible that IBS-D patients may relate to gut dysbiosis with decreased numbers of *Bacteroides*, *Bifidobacterium*, and/or *Lactobacillus*.

Our study has several limitations. First, this was a small study with limited sample size, which may not represent the entire existing gut microbiome. However, we performed both high-throughput sequencing and qPCR, which both demonstrated similar results and validates our findings of increased abundance of *Prevotella* in IBS-D patients. Second, we did not control for changes in diet between IBS patients and healthy controls. We know that long-term diet is one of the most critical factors in influencing the structure and composition of the gut microbiota [38]. However, our mouse model of PI-IBS also demonstrated increased abundance of *Prevotella*. These changes were not seen in the control mice even though they had identical diets.

In conclusion, we demonstrated that IBS-D in Chinese patients is closely associated with significant alterations in the gut microbiome that is characterized by reduced diversity and richness. Most significantly, we also discovered that enterotype enriched with *Prevotella* may be positively associated with high risk of IBS-D. Furthermore, we demonstrated that the altered intestinal microbiota is associated with visceral hypersensitivity in PI-IBS model.

## Figures and Tables

**Table 1:** Primers for qPCR.

Bacterium	Primer sequence (5′->3′)	Size (bp)
*Prevotella* species	F: CACCAAGGCGACGATCA	283
	R: GGATAACGCCCGGACCT	
*Bacteroides coli*	F: ATAGCCTTTCGAAAGAAAGAT	494
	R: CCAGTATCAACTGCAATTTTA	
*Bifidobacterium* species	F: GGGTGGTAATGCCGGATG	438
	R: TAAGCGATGGACTTTCACACC	
*Lactobacillus* species	F: AGCAGTAGGGAATCTTCCA	341
	R:CACCGCTACACATGGAG	

**Table 2:** The quantity of fecal bacteria in IBS-D patients and healthy controls.

Fecal bacteria	IBS-D (*N* = 40)	CON (*N* = 20)	*p* value
*Prevotella* spp.	7.91 ± 3.02	5.84 ± 1.82^∗^	*p* < 0.05
*Bacteroides*	8.99 ± 1.45	10.04 ± 1.00^∗∗^	*p* < 0.01
*Bifidobacterium* spp.	6.39 ± 1.14	7.21 ± 1.49^∗^	*p* < 0.05
*Lactobacillus* spp.	6.25 ± 0.98	6.94 ± 0.95^∗^	*p* < 0.05

Account unit is Log_10_ copies/g fecal ($\bar{x}\pm s$). Asterisks indicate statistical significance (^∗^*p* < 0.05, ^∗∗^*p* < 0.01 versus CON). CON represents for healthy controls.

**Table 3:** The quantity of fecal bacteria in PI-IBS and control mice.

Fecal bacteria	PI-IBS (*N* = 10)	CON (*N* = 4)	*p* value
*Prevotella* species	5.87 ± 0.40	5.31 ± 0.34^∗^	<0.05
*Bacteroides*	9.20 ± 0.74	8.36 ± 0.25	>0.05
*Bifidobacterium* species	5.44 ± 0.59	5.36 ± 0.49	>0.05
*Lactobacillus* species	4.87 ± 0.35	5.23 ± 1.16	>0.05

Account unit is Log_10_ copies/g fecal ($\bar{x}\pm s$). Asterisk indicates statistical significance (^∗^*p* < 0.05 versus CON). CON represents for normal mice.

**Table 4:** The quantity of fecal bacteria of mice in cohousing experiments.

Fecal bacteria	IBS-cohousing (*N* = 4)	CON-cohousing (*N* = 4)	*p* value (cohousing)	IBS-single (*N* = 5)	CON-single (*N* = 5)	*p* value (single)
*Prevotella* species	7.81 ± 1.34	7.55 ± 1.59	>0.05	8.51 ± 0.92	6.90 ± 0.69^∗^	<0.05
*Bacteroides*	10.93 ± 0.51	10.85 ± 0.24	>0.05	11.15 ± 0.52	10.58 ± 0.38	>0.05
*Bifidobacterium* species	5.50 ± 1.23	6.43 ± 0.69	>0.05	6.07 ± 0.95	6.08 ± 1.08	>0.05
*Lactobacillus* species	8.82 ± 0.40	9.03 ± 0.47	>0.05	9.41 ± 0.12	8.96 ± 0.92	>0.05

Account unit is Log_10_ copies/g fecal ($\bar{x}\pm s$). Asterisk indicates statistical significance (^∗^*p* < 0.05, CON-cohousing versus IBS-cohousing, CON-single versus IBS-single).

## Data Availability

The data used to support the findings of this study are included within the article.
